# The effect of pneumococcal immunization on total and antigen-specific B cells in patients with severe chronic kidney disease

**DOI:** 10.1186/s12865-019-0325-9

**Published:** 2019-11-12

**Authors:** Gabrielle Nicole Gaultier, William McCready, Marina Ulanova

**Affiliations:** 1 Department of Biology, Lakehead University, Thunder Bay, Canada; 2 Division of Medical Sciences, Northern Ontario School of Medicine, Lakehead University, 955 Oliver Road, Thunder Bay, ON P7B 5E1 Canada

**Keywords:** Chronic kidney disease (CKD), *Streptococcus pneumoniae*, B cells, Memory B cells, T-cell independent response, T-cell dependent response, 23-valent pneumococcal polysaccharide vaccine (PPV23), 13-valent pneumococcal conjugate vaccine (PCV13), Flow cytometry, Enzyme-linked immunospot assay (ELISPOT)

## Abstract

**Background:**

While the 23-valent pneumococcal polysaccharide vaccine (PPV23) is routinely used in Canada and some other countries to prevent pneumococcal infection in adults with chronic kidney disease (CKD), patients develop a suboptimal antibody response to PPV23 due to their immune dysfunction. The 13-valent pneumococcal conjugate vaccine (PCV13) has superior immunogenicity in some categories of immunocompromised adults; however, its effect on the immune response in CKD patients has only been addressed by two recent studies with conflicting results. The effect of PPV23 or PCV13 on B cells in these patients has not been previously studied. We studied the absolute numbers and proportions of B cells and subpopulations in two groups of adult patients with severe CKD pre- and 7 days post-immunization with PCV13: pneumococcal vaccine naïve and previously immunized with PPV23 (over one year ago).

**Results:**

PPV23 immunized patients had significantly lower proportions and absolute numbers of class switched memory (CD19 + CD27 + IgM-), as well as lower absolute numbers of IgM memory (CD19 + CD27 + IgM+) and class switched B cells (CD19 + CD27-IgM-) compared to PPV23 naïve patients. Following PCV13 immunization, the differences in absolute numbers of B-cell subpopulations between groups remained significant. The PPV23 immunized group had higher proportions of CD5- B cells along with lower proportions and absolute numbers of CD5+ B cells compared to PPV23 naïve patients both pre- and post-immunization with PCV13. However, previous PPV23 immunization did not have a noticeable effect on the numbers of total IgG or serotype 6B and 14 specific antibody-secreting cells detected 7 days post-immunization with PCV13. Nevertheless, fold increase in anti-serotype 14 IgG concentrations 28 days post-PCV13 was greater in PPV23 naïve than in previously immunized patients.

**Conclusions:**

The results suggest that immunization with PPV23 may result in long-term changes in B-cell subpopulations such as increased prevalence of CD5- B cells and decreased prevalence of class switched memory B cells in the peripheral blood. Because previous immunization with PPV23 in patients with CKD is associated with a significant decrease in the total class switched memory B cells in response to subsequent immunization with PCV13, this may reduce PCV13 immunogenicity in the setting of PPV23 followed by PCV13.

**Trial registration:**

Registered February 24, 2015 at ClinicalTrials.gov (NCT 02370069).

## Background

Chronic kidney disease (CKD) is a common condition which affects approximately 10–15% of adults globally [1]. Patients with the most advanced stages of this disease (severe CKD, or chronic renal failure) require life-saving renal replacement therapy, such as hemodialysis or kidney transplantation [2, 3]. Severe CKD is characterized by high mortality rates. Adjusted for age, sex, and race, the total mortality rate for patients with CKD as of 2018 was 103.0 per 1000 patient-years, more than double the total mortality rate for people without CKD [4]. Acute infections contribute substantially to the high rates of hospitalization and mortality in CKD patients, following only cardiovascular disease as a major cause of death [5]. High risk of septicaemia and other severe infections are attributed to both a compromised immune system and increased exposure to infectious agents in dialysis units [5–7].

Pneumococcus (*Streptococcus pneumoniae*) is a Gram-positive, encapsulated diplococcus, which commonly colonizes the upper respiratory tract [8]. Upon breaching the host defences, the microorganism can cause mucosal (otitis media, sinusitis, pneumonia) and invasive (meningitis, septicaemia, pericarditis, etc.) infections [9, 10]. *S. pneumoniae* is the most common cause of community acquired pneumonia (CAP) worldwide [11]. The highest incidence rates of CAP and invasive pneumococcal disease (IPD) are found in young children, elderly, and immunocompromised adults [9, 12]. Patients with CKD, particularly those with nephrotic syndrome and undergoing dialysis, are highly susceptible to pneumococcal infection, especially pneumonia [13].

To prevent pneumococcal infection in adult patients with CKD, immunization with pneumococcal polysaccharide vaccine (PPV23), which contains purified capsular polysaccharides from 23 pneumococcal serotypes most commonly associated with IPD (1, 2, 3, 4, 5, 6B, 7F, 8, 9 N, 9 V, 10A, 11A, 12F, 14, 15B, 17F, 18C, 19F, 19A, 20, 22F, 23F, and 33F), is currently recommended in Canada [14]. However, the effect of PPV23 in CKD patients is suboptimal because of their immune dysfunction [15, 16]. The second-generation (polysaccharide-protein conjugate) vaccines, which induce T-cell dependent antibody responses to polysaccharide antigens [17], have superior immunogenicity in immunocompromised adults and the elderly [18–20]. In Canada, 13-valent pneumococcal conjugate vaccine (PCV13), which consists of purified capsular polysaccharides of serotypes 1, 3, 4, 5, 6A, 6B, 7F, 9 V, 14, 18C, 19A, 19F, and 23F conjugated to a carrier protein (CRM_197_), is recommended for several categories of immunocompromised adults, such as bone marrow transplantation recipients and HIV-infected individuals [21]. Although in some countries PCV13 is now used for immunization of patients with CKD, PCV13 immunogenicity in adults undergoing hemodialysis was only recently addressed by two studies with differing results. Mitra et al. (2016) showed that the antibody concentrations in patients with CKD declined significantly 12 months after immunization with PCV13, compared to 2 months post-immunization for 11 of the 13 serotypes tested [22]. Vandecasteele et al. (2018) demonstrated that immunization of CKD patients with PPV23 may have a negative effect on the immune response to PCV13 [23]. It was suggested that immunization with PPV23 could lead to depletion of memory B cells following exposure to purified polysaccharide antigens potentially affecting the development of antibody responses to polysaccharide antigens administered via protein-conjugate vaccines [24]. However, to the best of our knowledge, the effect of PPV23 or PCV13 on B cells in patients with CKD has not been previously studied. To address this question, we studied the proportions and numbers of B cells (CD19+), and their subpopulations: naïve (CD27-IgM+), IgM memory (CD27 + IgM+), class switched (CD27-IgM-), class switched memory (CD27 + IgM-), CD5+ and CD5- B cells pre- and 7 days post-immunization with PCV13. Adult patients with severe CKD were separated into two groups: one group was pneumococcal vaccine naïve and the other group was previously immunized with PPV23 (over one year ago).

## Results

In patients with severe CKD, immunization with PCV13 did not result in any significant changes in proportions of total B cells or any B-cell subpopulation except for CD5- cells. The CD19 + CD5- subpopulation increased on day 7 post-immunization (74.2, 68.5–80.4% vs. 52.0, 39.3–68.7%, *p* <  0.05, Table 1). No significant changes in absolute numbers of total lymphocytes, B cells, or B-cell subpopulations assessed immediately prior to immunization and 7 days post-immunization were noticed (Additional file 1: Table S1). There was a statistically significant positive correlation between pre- and post-immunization absolute numbers of all tested cells, except for class switched and total B cells, and proportions of all cells with the exception of class switched and CD5- B cells (Additional file 2: Table S2).

**Table 1:** B-cell subpopulations in patients with severe chronic kidney disease

B cells	Pre-immunization % (CI)	Post-immunization % (CI)	p value
Total B cells (CD19+)	8.6 (7.0–10.6)	7.7 (6.9–8.5)	> 0.05
Naïve (CD27-IgM+)	46.3 (38.8–55.3)	42.5 (36.7–49.3)	> 0.05
IgM memory (CD27 + IgM+)	7.0 (5.2–9.5)	7.8 (6.5–9.4)	> 0.05
Class Switched (CD27-IgM-)	14.3 (12.6–16.3)	11.6 (10.1–13.3)	> 0.05
Class Switched memory (CD27 + IgM-)	17.6 (13.5–23.0)	21.4 (18–0-25.6)	> 0.05
CD19 + CD5+	30.2 (23.9–38.1)	24.9 (19.7–31.5)	> 0.05
CD19 + CD5-	52.0 (39.3–68.7)	74.2 (68.5–80.4)	< 0.05

Immunophenotype of peripheral blood B cells was determined by flow cytometry pre- and 7 days post-immunization with PCV13. Geometric mean (GM) of proportions with 95% confidence intervals (CI) are shown. For analysis of total B cells, naïve, IgM memory, class switched and class switched memory B cells, 33 pre-immunization and 60 post-immunization samples were studied. CD5 expression analysis, 33 pre-immunization and 39 post-immunization samples were studied. Statistical significance was determined by Mann-Whitney U test

**Fig. 1:**
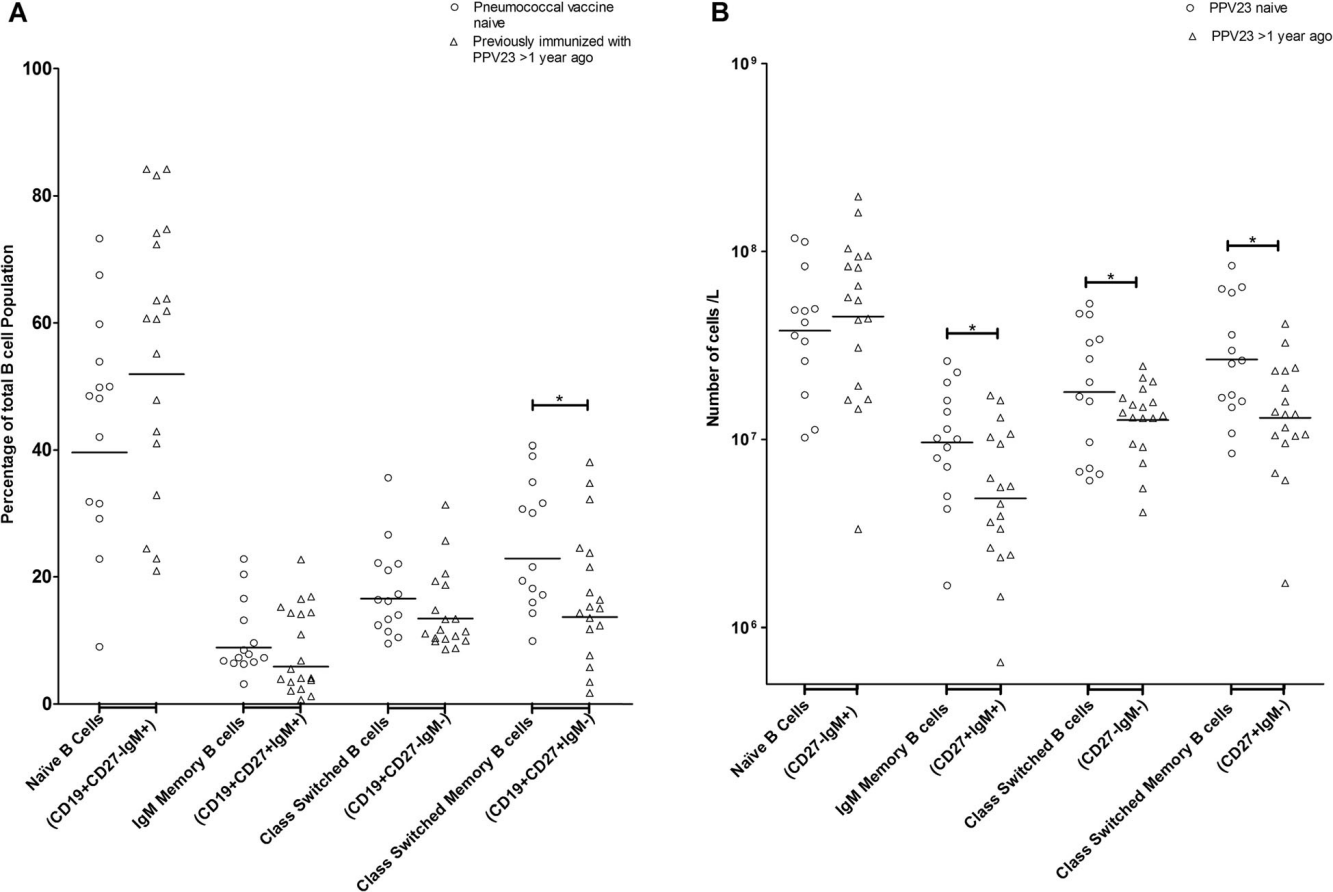
**a**. B-cell subpopulations in pneumococcal vaccine naïve (*n* = 14) and previously immunized with 23-valent pneumococcal polysaccharide vaccine (PPV23) (*n* = 19) patients with severe chronic kidney disease prior to immunization with 13-valent pneumococcal conjugate vaccine. Isolated peripheral blood mononuclear cells were stained for CD19, CD27, and IgM and analyzed by flow cytometry. The geometric mean of each subpopulation proportion is displayed. Statistical significance determined by Student’s t-test (*p < 0.05). **b**. Absolute numbers of B-cell subpopulations in pneumococcal vaccine naïve (n = 14) and previously immunized with 23-valent pneumococcal polysaccharide vaccine (PPV23) (n = 19) patients with severe chronic kidney disease prior to immunization with 13-valent pneumococcal conjugate vaccine. Isolated peripheral blood mononuclear cells were stained for CD19, CD27, and IgM. Geometric means of absolute numbers of B-cell subpopulations were determined by multiplying the proportions of B-cell subpopulations by the absolute number of lymphocytes. Statistical significance *p < 0.05 (Student’s t-test for IgM memory and class switched B cells, Mann-Whitney U test for class switched memory B cells)

To determine if PPV23 immunization had an impact on B-cell subpopulations, we tested the peripheral blood immediately prior to immunization with PCV13. The patients who had previously received PPV23 had significantly lower proportions (13.7, 9.2–20.3% vs. 22.9, 17.6–29.8, *p* <  0.05, Fig. 1A) and absolute numbers (1.3, 0.9–2.0 X 10^7^ cells/L vs. 2.6, 1.8–3.8 X 10^7^ cells/L, *p* < 0.05, Fig. 1b) of class switched memory B cells, as well as lower absolute numbers of IgM memory (4.9, 3.1–7.5 X 10^6^ cells/L vs. 9.6, 6.3–1.5 X 10^6^ cells/ L, p < 0.05) and class switched B cells (1.3, 1.0–1.6 X 10^7^ cells/L vs. 1.8, 1.8–4.1 X 10^7^ cells/L, p < 0.05) compared to PPV23 naïve patients (Fig. 1b). Previous immunization with PPV23 did not have a negative effect on total lymphocytes, as no significant difference in the absolute numbers of lymphocytes prior to PCV13 immunization was found between the groups (1.0, 0.8–1.4 X 10^9^ cells/ L vs. 0.9, 0.8–1.1 X 10^9^ cells/ L, *p* >  0.05, Additional file 3: Table S3). However, PPV23 naïve patients had a slightly higher absolute number of B cells pre-immunization compared to PPV23 immunized patients (9.9, 7.0–13.9 X 10^7^ cells/ L vs. 8.3, 6.1–11.2 X 10^7^ cells/ L, p >  0.05, Additional file 3: Table S3).

**Fig. 2:**
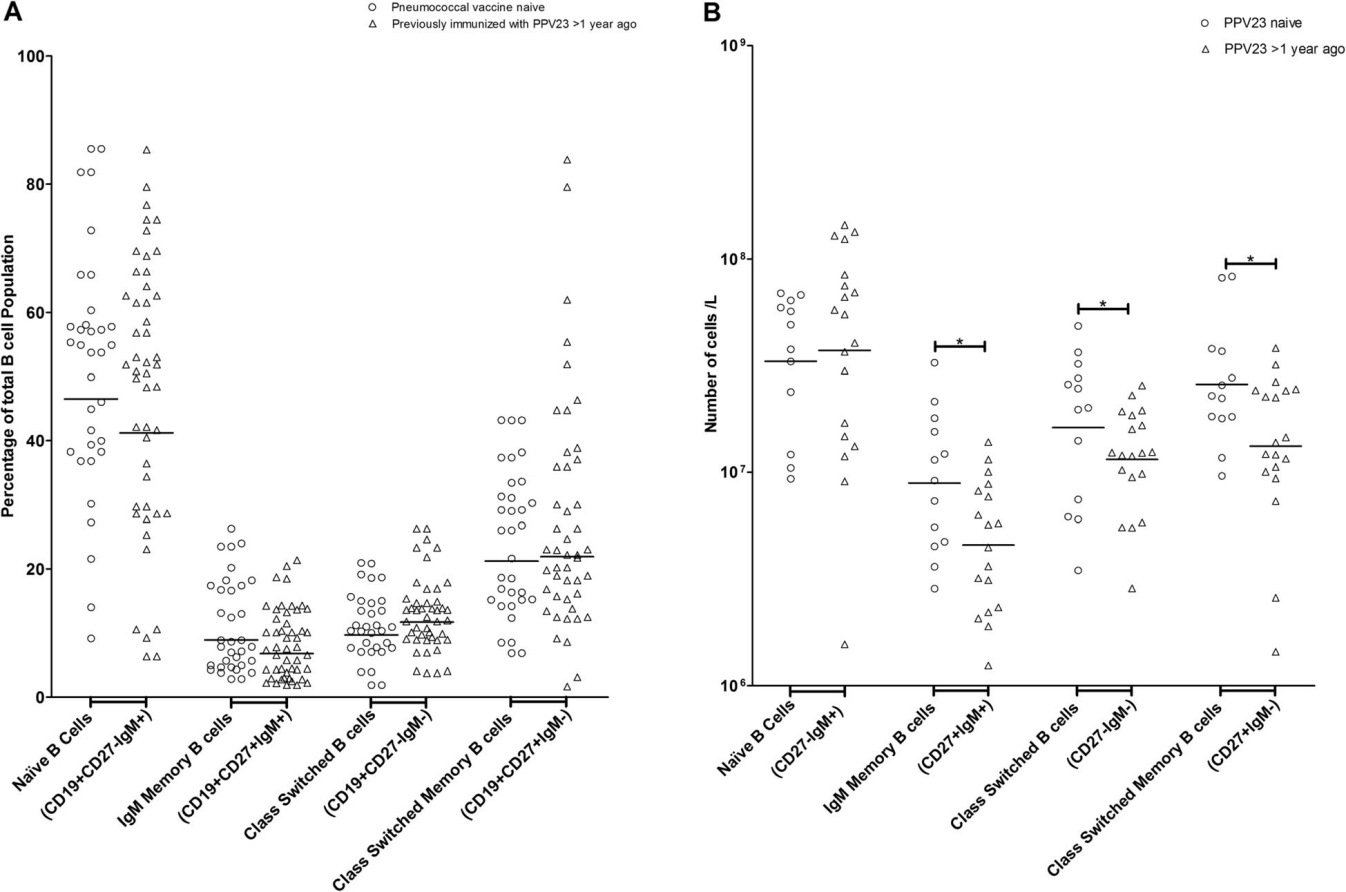
**a**. B-cell subpopulations in pneumococcal vaccine naïve (*n* = 25) and previously immunized with 23-valent pneumococcal polysaccharide vaccine (PPV23) (*n* = 35) patients with severe chronic kidney disease on day 7 post-immunization with 13-valent pneumococcal conjugate vaccine. Isolated peripheral blood mononuclear cells were stained for CD19, CD27, and IgM and analyzed by flow cytometry. The geometric mean of each subpopulation proportion is displayed. No statistically significant difference was detected between the groups. **b**. Absolute numbers of B-cell subpopulations in pneumococcal vaccine naïve (n = 14) and previously immunized with 23-valent pneumococcal polysaccharide vaccine (PPV23) (n = 19) patients with severe chronic kidney disease 7 days post-immunization with 13-valent pneumococcal conjugate vaccine. Isolated peripheral blood mononuclear cells were stained for CD19, CD27, and IgM. Geometric mean absolute numbers of B-cell subpopulations were determined by multiplying the proportions of B-cell subpopulations by the absolute number of lymphocytes. Statistical significance *p < 0.05 (Student’s t-test for class switched B cells, Mann-Whitney U test for IgM memory and class switched memory B cells)

Following the PCV13 immunization, the differences in absolute numbers of B-cell subpopulations between the groups remained significant. Those who had previously received PPV23 had lower numbers of IgM memory (4.6, 3.2–6.5 X 10^6^ cells/ L vs. 8.9, 5.7–14.0 X 10^6^ cells/ L, *p* < 0.05), class switched B cells (1.2, 0.9–1.5 X 10^7^ cells/ L vs. 1.6, 1.0–2.7 X 10^7^ cells/ L, p < 0.05), and class switched memory B cells (1.3, 0.9–2.0 X 10^7^ cells/ L vs. 2.6, 1.8–3.8 X 10^7^ cells/ L, p < 0.05) compared to PPV23 naïve patients (Fig. 2b), although no significant differences in proportions of B-cell subpopulations between the groups were detected (Fig. 2a). Both groups had the same absolute numbers of lymphocytes 7 days post-immunization (1.0, 0.7–1.3 X 10^9^ cells/ L vs. 1.0, 0.8–1.2 X 10^9^ cells/ L, *p* >  0.05, Additional file 3: Table S3). However, PPV23 naïve patients had slightly higher absolute numbers of total B cells compared to PPV23 immunized patients (9.3, 6.7–12.8 X 10^7^ cells/ L vs. 7.9, 5.7–10.9 X 10^7^ cells/ L, p >  0.05, Additional file 3: Table S3). The results suggest that previous immunization with PPV23 may decrease the numbers of IgM memory, class switched, and class switched memory B-cell subpopulations both pre- and 7 days post-immunization with PCV13.

**Fig. 3:**
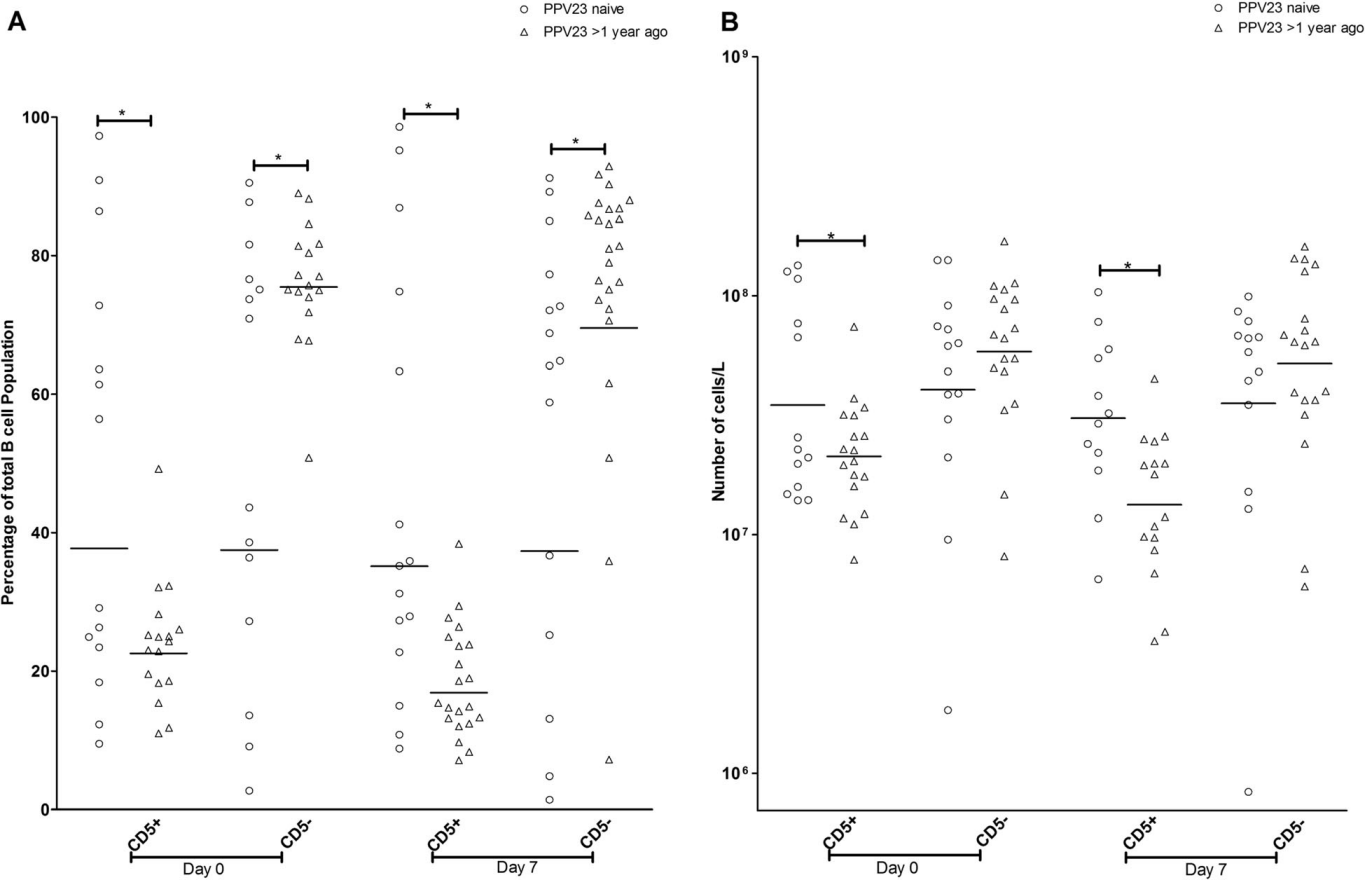
**a**. CD5+ and CD5- B-cell subpopulations pre-immunization (day 0) and post-immunization (day 7) with 13-valent pneumococcal conjugate vaccine in patients with severe chronic kidney disease. Peripheral blood mononuclear cells were stained for CD19 and CD5 and analyzed by flow cytometry. The geometric means are displayed. CD5+ and CD5- B-cell subpopulations are compared between pneumococcal vaccine naïve (day 0, n = 14; day 7, *n* = 15) and previously immunized with 23-valent pneumococcal polysaccharide vaccine (PPV23) (day 0, n = 19; day 7, *n* = 24) patients. Statistical significance *p < 0.05 (Mann-Whiney U test for day 0 CD5+, CD5- and day 7 CD5- B cells, Student’s t-test for day 7 CD5+ B cells). **b**. Absolute numbers of CD5+ and CD5- B-cell subpopulations pre-immunization (day 0) and post-immunization (day 7) with 13-valent pneumococcal conjugate vaccine in patients with severe chronic kidney disease. Absolute numbers of B-cell subpopulations were determined by multiplying the proportions of B-cell subpopulations by the absolute number of lymphocytes. The geometric means are displayed. Comparison between pneumococcal vaccine naïve (day 0, n = 14; day 7, n = 15) and previously immunized with 23-valent pneumococcal polysaccharide vaccine (PPV23) (day 0, *n* = 19; day 7, n = 24) patients is displayed. Statistical significance *p < 0.05 (Student’s t-test for day 0 CD5+ B cells, Mann-Whitney U test for day 7 CD5+ B cells)

As previous studies found an association between the CD5- subpopulation of B cells and production of IgG antibodies to pneumococcal polysaccharide antigens in response to PPV23 [25], we attempted to determine if there were any differences in CD5-expressing B cells between the groups with different histories of PPV23 vaccination. Prior to immunization with PCV13, those patients who had previously been immunized with PPV23 had higher proportion of CD5- (75.5, 70.6–80.6% vs. 37.5, 20.6–68.3%, *p* < 0.05) and lower proportion of CD5+ cells (22.5, 18.9–27.2% vs. 37.7, 24.1–58.86%, p < 0.05) as well as CD5+ absolute numbers (2.1, 1.6–2.8 X 10^7^ vs. 3.5, 2.0–6.0 X 10^7^, p < 0.05) compared to PPV23 naïve patients (Fig. 3a, b).

Seven days post-immunization with PCV13, patients previously immunized with PPV23 still had higher proportions of CD5- B cells (69.5, 55.7–86.9% vs. 37.4, 18.9–73.66%, p < 0.05) and lower proportions (16.9, 13.9–20.6% vs. 35.2, 23.0–53.7%, p < 0.05) and absolute numbers of CD5+ cells (3.0, 1.9–5.1 X 10^7^ cells/ L vs. 1.3, 0.9–1.9 X 10^7^ cells/ L, p < 0.05) compared to PPV23 naïve patients (Fig. 3a, b). Although higher absolute numbers of CD5- cells were present in PPV23 immunized than in PPV23 naive patients, the difference was not statistically significant (5.2, 3.3–8.1 X 10^7^ cells/ L vs. 3.5, 1.6–7.7 X 10^7^ cells/ L, *p* >  0.05, Fig. 3b). These results show that immunization with PPV23 may lead to in an increased prevalence of the CD5- subpopulation among circulating B cells.

**Fig. 4:**
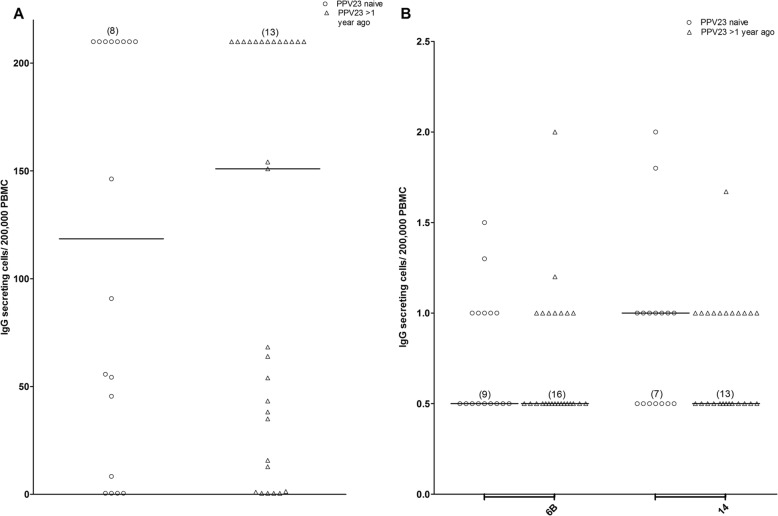
**a**. Numbers of total IgG antibody secreting cells (ASC) per 200,000 peripheral blood mononuclear cells (PBMC) pneumococcal vaccine naïve (*n* = 18) and previously immunized with 23-valent pneumococcal polysaccharide vaccine (PPV23) (*n* = 29) patients with severe chronic kidney disease on day 7 post-immunization with PCV13. The median is displayed counts above the upper limit of detection (200) were assigned a value of 210 (8 pneumococcal vaccine naïve and 13 previously immunized with PPV23). If no spots were detected, a value of 0.5 was assigned for statistical purposes. PPV23 naïve and PPV23 immunized each had 4 values below the limit of detection. Control wells coated with methylated human serum albumin were not displayed (all values were below the limit of detection). **b**. Numbers of IgG antibody secreting cells (ASC) specific for pneumococcal capsular polysaccharides of serotypes 6B or 14 per 200,000 peripheral blood mononuclear cell (PBMC) in pneumococcal vaccine naïve (n = 18) and previously immunized with 23-valent pneumococcal polysaccharide vaccine (PPV23) (*n* = 31) patients with severe chronic kidney disease 7 days post-immunization with PCV13 (median is shown). If no spots were detected a value of 0.5 was assigned for statistical purposes. For serotype 6B, 9 pneumococcal vaccine naïve and 16 previously immunized with PPV23 patients had ASC below the lower limit of detection. For serotype 14, 7 pneumococcal vaccine naïve and 13 previously immunized with PPV23 patients had ASC below the lower limit of detection. Control wells coated with methylated human serum albumin were not displayed (all below the limit of detection)

To determine if the numbers of total IgG and antigen specific antibody secreting cells (ASC) on day 7 post-immunization with PCV13 depend on previous immunization with PPV23, we conducted enzyme-linked immunospot (ELISPOT) assay on peripheral blood mononuclear cells (PBMC) stimulated with *Staphylococcus aureus* Cowan strain protein A (SAC) and CpG Oligonucleotide (ODN-2006). No statistically significant difference between PPV23 naïve and PPV23 immunized patients was found between the numbers of total IgG ASC (median ± standard deviation) (118.5 ± 93.8 vs. 151.0 ± 93.5, *p* >  0.05, Fig. 4a), ASC specific for pneumococcal polysaccharide serotype 6B (0.5 ± 0.34 ASC vs. 0.5 ± 0.36 ASC), or serotype 14 (1.0 ± 0.5 ASC vs. 0.8 ± 0.3 ASC, *p* >  0.05, Fig. 4b). After outliers were removed, the number of patients that had antigen-specific IgG ASC below the limit of detection was calculated. There were 9/16 (56%) PPV23 naïve and 16/25 (64%) PPV23 immunized patients without detectable ASC specific for 6B; for serotype 14-specific ASC, the corresponding numbers were 7/16 (44%) vs.13/25 (52%), *p* >  0.05 (Fisher’s exact test). Hence, in this group of patients, previous PPV23 immunization did not have a noticeable effect on the numbers of total IgG ASC or serotype 6B and 14 specific ASC detected at day 7 post-immunization with PCV13.

To determine if previous immunization with PPV23 had an effect on the antibody response to PCV13, concentrations of pneumococcal 6B and 14 IgG antibodies were determined pre- and 28 days post-immunization (Additional file 4: Table S4). There were no significant differences between PPV23 naive and PPV23 immunized patients in the concentration of pneumococcal 6B antibodies pre-immunization (0.9, 0.6–1.4 μg/ mL vs. 1.1, 0.7–1.6 μg/ mL, *p* >  0.05) or post-immunization (2.1, 1.4–3.2 μg/ mL vs. 2.4, 1.4–3.9 μg/ mL, p >  0.05). Compared to PPV23 immunized patients, PPV23 naive patients had a significantly lower concentration of pneumococcal 14 antibodies pre-immunization (2.1, 1.4–3.1 μg/ mL vs. 2.4, 1.4–3.9 μg/ mL, *p* < 0.05), but there was no significant difference in post-immunization concentrations (8.0, 4.5–14.4 μg/ mL vs. 8.1, 5.0–14.4, *p* > 0.05). When pre- and day 28 post-immunization concentrations were compared for each patient group, both groups had a significant increase in pneumococcal 6B antibody concentrations (p < 0.05), however, only PPV23 naïve patients had a significant increase in pneumococcal 14 antibody concentrations (*p* < 0.01).

While PPV23 immunized patients had a slightly smaller fold change in pneumococcal 6B antibodies compared to PPV23 naïve patients (2.3, 1.7–3.1 μg/ mL vs. 2.8, 1.8–4.3 μg/ mL, p > 0.05), they had a significantly smaller fold change in pneumococcal 14 antibodies (1.3, 1.1–1.6 μg/ mL vs. 1.9, 1.3–2.8 μg/ mL, p < 0.05). A strong negative correlation was found between the fold change of pneumococcal 14 antibodies and the pre-immunization absolute numbers of CD5- B cells in PPV23 immunized patients (r = − 0.8576, *p* < 0.001, Pearson correlation). No other significant correlation was detected between pre-immunization or day 7 post-immunization proportions or absolute numbers of CD5- or class switched memory B-cell subpopulations and the fold change in pneumococcal 6B or 14 antibody concentrations (data not shown). These results suggest that previous PPV23 immunization negatively affects the pneumococcal 6B and 14 antibody response to subsequent immunization with PCV13 in patients with CKD.

## Discussion

Although both types of pneumococcal vaccines induce the production of serotype-specific antibodies to *S. pneumoniae* capsular polysaccharides, PPV23 and PCV13 activate different immunological mechanisms. PPV23 contains purified capsular polysaccharides, which directly activate B cells in a T-cell independent manner. Bacterial capsular polysaccharides have multiple repeating epitopes which cross-link B cell receptors, causing the activation of Bruton’s tyrosine kinase that results in activation and proliferation of antigen-specific B cells [26]. The memory B cells generated from T-cell independent responses are short-lived and produce a less robust antibody response upon re-exposure to an antigen [27]. Vaccines containing capsular polysaccharides alone, such as PPV23, have poor immunogenicity in immunocompromised individuals [28]. In the case of PCV13, the conjugation of polysaccharides to CRM_197_, a non-toxic mutant of diphtheria toxin, which acts as a carrier protein, results in a T-cell dependent response. The protein-polysaccharide conjugate binds to B cell receptors and it is then brought into the endosome, where the protein component is processed into peptides and binds to the major histocompatibility complex class II (MHC II) molecules. Antigen presentation of the carrier protein in the context of MHC II results in the activation of CD4+ T cells. The subsequent generation of signals stimulates B cell maturation, class switching, and proliferation resulting in the production of long-lived memory B cells and secretion of polysaccharide-specific class switched high affinity antibodies [29].

It is still uncertain whether immunization with plain pneumococcal polysaccharide antigens (included into PPV23) negatively impacts antibody responses to subsequent immunization with polysaccharide-protein conjugate vaccines, such as PCV13. A study by Clutterbuck et al. (2012) measured the effect of PPV23 followed by the 7-valent pneumococcal conjugate vaccine (PCV7) on antigen-specific B cells in older adults. It was found that while PCV7 immunization resulted in an increase in pneumococcal serotype-specific memory B cells, immunization with PPV23 depleted these cells and resulted in attenuated memory B cell responses [24]. No studies have assessed the effect of pneumococcal immunizations on B cells in patients with CKD. Vandecasteele et al. (2018) found that severe CKD patients previously immunized with PPV23 had lower antibody response to PCV13 compared to pneumococcal vaccine naïve patients [23]. Our results agree with this study, as PPV23 immunized patients had a slightly lower fold change in pneumococcal 6B IgG antibodies, and significantly lower fold change in pneumococcal 14 antibodies in response to PCV13 immunization. Recent studies assessed the effect of previous PPV23 immunization on the response to PCV13 or PCV7 in older adults found that previous immunization with PPV23 decreased antibody response to subsequent doses of PCV13 [20, 30, 31].

Patients with CKD are unique among other high-risk groups because they have multiple predisposing factors to pneumococcal infection, including uremia with its significant metabolic consequences, as well as the hemodialysis procedure, which causes premature aging of the immune system [6, 32]. Patients with CKD also frequently have comorbidities, such as diabetes mellitus, which contribute to their immune dysfunction, and may receive immunosuppressive medications [33]. These patients also have increased exposure to infectious agents in the hospital environment. Multiple immune abnormalities such as decreased bactericidal ability of neutrophils, granulocyte and macrophage phagocytic function, defective function of antigen presenting cells, reduced numbers of B cells and decreased antibody producing capacity of plasma cells, increased T- and B-cell apoptosis, decreased thymic T-cell output, and impaired activation of T-cell response have been found in CKD patients [34, 35].

Among immune abnormalities, this group has noticeable alterations in B cells and B-cell function. We have recently found that patients with CKD had decreased absolute numbers of B cells and B-cell subpopulations compared to healthy controls [36]. Pahl et al. (2010) also found decreased numbers of B cells in CKD patients, suggesting that B-cell lymphopenia in patients with CKD was due to uremia [37]. It is possible that epigenetic changes caused by CKD affect the production of lymphoid cells at the hematopoietic stem cell level, resulting in decreased numbers of circulating B and T cells [38, 39].

In this study, we found that immunization with PPV23 may have a long-term effect on B-cell subpopulations in patients with CKD. Compared to vaccine naïve patients, PPV23 immunized patients of a similar age had significantly lower proportions of class switched memory B cells pre-immunization, and significantly lower absolute numbers of these cells both pre- and 7 days post-immunization with PCV13. In the literature, class switched memory B cells are defined as CD19 + CD27 + IgD- or CD19 + CD27 + IgM- cells [40, 41]. These cells respond rapidly when re-exposed to the same antigen, resulting in the production of class switched, high affinity antibodies, and hence mediate long-lived humoral immunity [42, 43].

Although pneumococcal-specific B cells are only a small proportion of the total B cell population (0.5% of the total B cell population pre-immunization and 2.0–2.75% 7 days post-immunization with PPV23) [41], our results suggest that immunization with PPV23 results in a significant decrease of total class switched memory B cell population. This has not been previously reported as most studies focus on antigen-specific memory B cells. Chovancova et al. (2011) did not detect a significant change in the proportion or absolute number of class switched memory B cells defined as CD19 + CD27 + IgD-, between pre- and 7 days post-immunization with PPV23 in adults with common variable immunodeficiency (CVID) [44]. It is possible that immunization with pneumococcal polysaccharides results in polyclonal stimulation of B cells, leading to terminal differentiation of memory B cells followed by their depletion. Polyclonal stimulation of memory B cells, which causes their proliferation and differentiation into plasma cells, helps to maintain serological memory throughout life [45]. Polyclonal B cell activation by capsular polysaccharides of *Neisseria meningitidis* was previously reported by Oliveira et al. (1996) [46].

The role of CD5+ and CD5- B cells in humans is not completely understood. Due to the lack of somatic hypermutations in CD5+ B cells, it is thought that these cells are naïve B cells [25, 47]. The expression of CD5 decreases with age and is associated with an increase in CD27 expression, indicating a shift in B-cell subpopulations towards an increase in the population of memory B cells [48]. A study by Moens et al. (2015) determined that after immunization with PPV23, ASC originating from CD5- B cells primarily produce pneumococcal anti-capsular polysaccharide IgG antibodies [25]. In agreement with this study, we found significantly higher proportions of CD5- B cells in PPV23 immunized patients compared to PPV23 naïve ones. Hence immunization with PPV23 favors the generation of CD5- pneumococcal IgG memory B cells even in these immunocompromised adults. We found a strong negative correlation between the pre-immunization absolute numbers of CD5- B cells and the fold change of pneumococcal 14 IgG antibodies in PPV23 immunized patients. Hence, although previous immunization with PPV23 favours the generation of CD5- B cells, it has a negative effect on the antibody response to subsequent immunization with PCV13 in patients with CKD.

In an attempt to assess antigen-specific ASC, we used PBMC collected 7 days post immunization, given that ASC can be detected in the peripheral blood as early as 5 days after vaccination or infection and their numbers peak at day 7 [49]. The cells were stimulated with B-cell polyclonal activators, i.e. SAC, which induces crosslinking of surface immunoglobulins [50] and CpG ODN-2006, which activates TLR 9 that stimulate the differentiation of memory B cells [51]. We measured the B-cell responses to pneumococcal capsular polysaccharides of serotypes 6B and 14 because they have a significant impact on the global epidemiology of IPD [52].

As we detected only small numbers of ASC, this may reflect poor functional ability of B cells from CKD patients to respond to polyclonal stimulation. In addition, capsular polysaccharides of serotypes 6B and 14 have low immunogenicity [52]. As various modifications of ELISPOT exist, including different methods of B cell stimulation, it is difficult to compare our results to other studies. One study determined that the more doses of PPV23 asplenic adults with β-thalassemia received, the lower the amount of capsular polysaccharide-specific memory B cells in response to PCV13 was detected [53]. They also found that longer interval between PPV23 and PCV13 vaccinations was associated with greater numbers of antigen-specific memory B cells [53]. In that study, the range of capsular polysaccharide-specific IgG ASC per 1,000,000 PBMC was 0–24; in comparison, our patients had a maximum of two ASC specific to 6B and 14 per 200,000 PBMC. Such discrepancies could be due to differences in age and immune abnormalities of participants. When we attempted to measure numbers of plasma cells using ELISPOT, no plasma cells specific for 6B or 14 were detected (data not shown). Similarly, in the study by Chovancova et al. (2011), CVID patients had no detectable IgG ASC specific for 23 pneumococcal capsular polysaccharides due to the lack of terminal B cell differentiation [44]. This could potentially explain the lack of B-cell response in CKD patients as it was demonstrated that such patients have decreased B cell activating factor receptor expression in transitional B cells, resulting in their decreased survival [37].

Our study has several limitations, including small sample size that could contribute to lack of statistical significance in comparisons between groups. The patients received PPV23 at different times prior to PCV13 immunization (although always over 1 year before) that could contribute to the variability in results; we were unable to measure their immediate response to the PPV23 vaccine. Due to the large diversity among the CKD patients, we could not match our study participants for underlying conditions. We did not have healthy aged-matched controls that would have helped in the interpretation of responses by CKD patient groups. We were unable to calculate the fold change in antibody concentration for all participants due to sample collection issues such as participant withdrawal, etc. Our ongoing study of antigen-specific antibody responses in these two groups will aid in the clarification of the effect of previous immunization with PPV23 on B cells and their ability to respond to subsequent immunization with PCV13. In addition, our work will help determine the longevity of the antibody response to PCV13 in patients with CKD.

## Conclusions

Immunization with PPV23 may result in long-term changes in B-cell subpopulations such as an increased prevalence of CD5- B cells and decreased prevalence of class switched memory B cells in the peripheral blood. Because previous immunization with PPV23 in patients with CKD is associated with a significant decrease in the total class switched memory B cell population as well as a decreased pneumococcal antibody response to subsequent immunization with PCV13, this may reduce PCV13 immunogenicity in the setting of PPV23 followed by PCV13. Our findings emphasize the need for further studies to optimize pneumococcal immunization for adults with CKD.

## Methods

### Patient recruitment and eligibility

Sixty-one patients with stage 4 or 5 severe CKD (glomerular filtration rate (GFR), stage 4 < 30 mL/ min/ 1.73m^2^, stage 5 < 15 mL/ min/ 1.73m^2^) [54] who were receiving hemodialysis at the Thunder Bay Regional Health Sciences Centre (TBRHSC) were recruited between May 2015 and August 2018. All the patients were over 18 years old, did not have a history of immunocompromising conditions, were not taking any immunosuppressive medications for more than 14 days in the past 6 months, and had not received any vaccines in the past month or blood transfusions in the past 3 months. Patients were separated into two groups: 25 PPV23 naïve patients (mean age 59 years, range 20–87 years, 32% female) and 36 previously immunized with PPV23 > 1 year ago (mean age 60 years, range 32–87 years, 47% female). This study was registered at ClinicalTrials.gov (NCT 02370069) and approved by the research ethics boards of Lakehead University and TBRHSC.

### Immunization protocol and sample collection

All patients received a single dose of PCV13 (Prevnar13, Pfizer, lots: H22520, J77978, M573833, M73833, R56653, and T24279) administered intramuscularly in the deltoid region during the dialysis procedure. Ten milliliters of peripheral blood for B-cell analysis were collected into BD Vacutainer™ tubes with sodium heparin (BD Biosciences, Baltimore, MD, USA) immediately prior to immunization for flow cytometry analysis and 7 days post-immunization for flow cytometry and ELISPOT. Blood was collected for complete blood counts (CBC) just before immunization and day 7 post-immunization. An additional 10 mL of peripheral blood for serum antibody analysis was collected into BD Vacutainer™ Venous Blood Collection Tubes: SST™ Serum Separation Tubes: Hemogard (BD Biosciences) pre- and 28 days post-immunization, serum was stored at − 80 °C.

### Analysis of B cells

PBMC were isolated by density gradient centrifugation using Lymphoprep (Stemcell Technologies, Vancouver, BC, CAN). Monocytes were removed using two consecutive incubations in RPMI 1640 medium with L-glutamine (Thermo Fisher Scientific, Mississauga, ON, CAN) supplemented with 1% antibiotic-antimicotic (Life Technologies, Burlington, ON, CAN) and 20% fetal bovine serum (FBS, Fisher Scientific, Whitby, ON, CAN) (20% supplemented medium) in a BD Falcon™ 100 X 20 mm cell culture dish (Fisher Scientific) for 1 h at 37 °C, 5% CO_2_. Non-adherent cells were washed with RPMI 1640 medium with L-glutamine supplemented with 1% antibiotic-antimicotic and 10% FBS (10% supplemented medium) and re-suspended at 2 X 10^6^ cells/ mL in 10% supplemented medium. From the cell suspension, 200,000 cells were immunostained with PE Mouse Anti-Human CD19, APC Mouse Anti-Human IgM, PerCP-Cy™5.5 Mouse Anti-Human CD27, and FITC Mouse Anti-Human CD5 (BD Biosciences) at 4 °C for 1 h. Samples were analyzed with BD FACSCalibur™ Flow Cytometer and CELLQUEST PRO software (BD Biosciences) to determine proportions of B cells (CD19+) and subpopulations: naïve (CD27-IgM+), IgM memory (CD27 + IgM+), class switched (CD27-IgM-), class switched memory (CD27 + IgM-), CD5+ and CD5- B cells as previously described [36]. Purity of CD19+ gated B cells was verified by counterstaining cells with FITC Mouse Anti-Human CD3 and PerCP-Cy™5.5 Mouse Anti-Human CD14 (BD Biosciences).

To determine the absolute numbers of B cells, a CBC was performed at the TBRHSC clinical lab. The absolute number of B cells was calculated by multiplying the percentage of CD19+ cells of the total gated lymphocyte population by the total lymphocyte count. The numbers of B cell subpopulations were determined by multiplying the percentage of each subpopulation by the absolute number of B cells.

### Enzyme-linked immunospot (ELISPOT) assay

#### Preparation of ELISPOT plates

Multi-screen IP 96-well PVDF membrane filter plates (Millipore Canada Ltd., Etobicoke, ON, CAN) were coated with either goat anti-human IgG (20 μg/ mL) (Cedarlane, Burlington, ON, CAN), or pneumococcal capsular polysaccharides of serotypes 6B, or 14 (10 μg/ mL) (Cedarlane) conjugated with methylated human serum albumin (mHSA, 10 μg/ mL) (National Institute for Biological Standards and Control, Hertfordshire, UK). Protocol for conjugation of the pneumococcal polysaccharides to mHSA for coating plates was provided by the Oxford Vaccine Group. Wells containing solution of mHSA (10 μg/ mL) in PBS (Fisher Scientific) served as “no coating control”. Plates were incubated at 37 °C, 5% CO_2_ for 5 h then stored at 4 °C overnight. The next day, plates were washed with PBS then blocked with 2% skim milk (Thermo Fisher Scientific) in PBS for 3 h at room temperature. The plate was washed again with PBS before cells were added.

### Detection of memory B cells

Following removal of monocytes, 2 X 10^6^ PBMC were plated in a 6-well plate in 10% supplemented medium with SAC (5 μg/ mL) (Sigma Aldrich, Oakville ON, CAN) ODN-2006 (3 μg/ mL) (Cedarlane, Burlington, ON, CAN). After 6 days of stimulation at 37 °C, 5% CO_2_, the cells were harvested, washed with 10% supplemented medium, re-suspended in the same medium and counted. Cells were plated at 200,000 cells per well in the 96-well coated plate at 37 °C, 5% CO_2_ for 18 h. After the incubation, the plate was washed using PBS with 0.01% Tween 20 (Fisher Scientific) before the HRP conjugated mouse anti-human IgG was added (0.13 μg/ mL) (Hybridoma Reagent Laboratory, Baltimore, MD, USA) for 2 h at room temperature. To visualize the spots, KPL TrueBlue™ Peroxidase Substrate (Mandel Scientific, Guelph, ON, CAN) was added for 10 min and then washed for 5 min with distilled water. After washing, the plate was left to dry for a minimum of 1 day. Once dry, the spots were visualized using 2X magnification (Nikon Eclipse 80i).

### ELISPOT counting

To determine the numbers of ASC, two lab members counted the spots per well individually, and a mean of the counts was calculated. Counts over 200 were considered above detection limits and assigned a value of 210; counts of 0 were assigned a value of 0.5 for statistical purposes.

#### Enzyme-linked immunosorbent assay (ELISA)

Amounts of IgG anti-pneumococcal 6B and 14 antibodies were quantified using the ELISA protocol provided by the World Health Organization [55].

#### Statistical analysis

Statistical analysis was performed using Graph-Pad Prism 5 (GraphPad Prism Software Inc., San Diego, CA). The geometric means for the percentage of each B-cell subpopulation, absolute numbers of B cells, concentration, and fold change of antibody concentration with 95% confidence intervals (CI) and the medians of ASC with standard deviation were calculated. Outliers were identified and removed prior to statistical analysis [56]. Groups were compared either using a Student’s t-test or Mann-Whitney U test, and correlation analyses performed using Pearson or Spearman analysis based on the distribution of the data. A *p* value of < 0.05 was reported as statistically significant.

## Supplementary information

**Additional file 1:** **Table S1.** Absolute numbers of lymphocytes and B cells in patients with severe chronic kidney disease. Absolute numbers of lymphocytes and B cells (geometric means, GM with 95% confidence intervals, CI) in 33 patients with severe chronic kidney disease pre- and 7 days post-immunization with PCV13. Absolute numbers of B cells were determined by multiplying the proportion of CD19+ peripheral blood mononuclear cells by the absolute number of lymphocytes obtained via complete blood counts.

**Additional file 2:** **Table S2.** Correlation of absolute numbers and proportions of B cells and B-cell subpopulations pre- and 7 days post-immunization. Correlation between pre- and post-immunization absolute numbers or proportions of total B cells (CD19+), naïve (CD27-IgM+), IgM memory (CD27 + IgM+), class switched (CD27-IgM-), class switched memory (CD27 + IgM), CD5+ and CD5- B cells.

**Additional file 3:** **Table S3.** Absolute numbers of lymphocytes and B cells in patients with severe chronic kidney disease that are pneumococcal vaccine naïve or previously immunized with PPV23 > 1 year ago. Absolute numbers of lymphocytes and B cells (geometric means, GM with 95% confidence intervals, CI) in severe chronic kidney disease patients that are pneumococcal vaccine naïve (n = 14) or previously immunized with PPV23 > 1 year ago (n = 19) pre- and 7 days post-immunization with PCV13.

**Additional file 4: Table S4.** Concentrations and fold change of pneumococcal 6B and 14 IgG antibodies pre- and 28 days post-immunization. Geometric mean concentration (GMC) with 95% confidence intervals (CI) in severe chronic kidney disease patients who are pneumococcal vaccine naïve (*n* = 22) or previously immunized with PPV23 > 1 year ago (*n* = 34) pre- and 28 days post-immunization with PCV13. The fold change represents the response to PCV13 immunization. * compares day 0 pre-immunization and day 28 post-immunization antibody concentrations for each serotype and patient group, + compares the pre-immunization concentrations between patient groups, # compares the fold change for each serotype between groups. Statistical significance determined by Mann- Whitney U test, * p < 0.05, ** *p* < 0.01.

## Data Availability

The datasets used and/or analysed during the current study are available from the corresponding author on reasonable request.

## Abbreviations

ASC: antibody secreting cells
CBC: complete blood count
CI: confidence intervals
CKD: chronic kidney disease
ELISA: enzyme-linked immunosorbent assay
ELISPOT: Enzyme-linked immunospot assay
MHC II: Major histocompatibility complex class II
mHSA: methylated human serum albumin
ODN: CpG Oligonucleotide (ODN-2006)
PBMC: Peripheral blood mononuclear cells
PCV13: 13-valent pneumococcal conjugate vaccine
PCV7: 7-valent pneumococcal conjugate vaccine
PPV23: 23-valent pneumococcal polysaccharide vaccine
SAC: *Staphylococcus aureus* Cowan strain protein A
