# Supplementary material for: The effect of pneumococcal immunization on total and antigen-specific B cells in patients with severe chronic kidney disease
Source: BMC Immunol. 2019 Nov 12;20:41. doi: 10.1186/s12865-019-0325-9 (PMC6849264; doi:10.1186/s12865-019-0325-9)
Supplement: Supplementary file 1 — Additional file 1: Table S1. Absolute numbers of lymphocytes and B cells in patients with severe chronic kidney disease. Absolute numbers of lymphocytes and B cells (geometric means, GM with 95% confidence intervals, CI) in 33 patients with severe chronic kidney disease pre- and 7 days post-immunization with PCV13. Absolute numbers of B cells were determined by multiplying the proportion of CD19+ peripheral blood mononuclear cells by the absolute number of lymphocytes obtained via complete blood counts. [file 12865_2019_325_MOESM1_ESM.docx]

| Cells | Pre-immunization  Absolute number of cells/L (GM, CI) | 7 days post-immunization  Absolute number of cells/L (GM, CI) | p value |
| --- | --- | --- | --- |
| Lymphocytes | 1.0 (0.8-1.4 X 10^9^) | 1.0 (0.8-1.1 X 10^9^) | > 0.05 |
| Total B cells | 9.2 (7.4-11.5 X 10^7^) | 8.4 (6.7-10.5 X 10^7^) | > 0.05 |
| Naïve (CD27-IgM+) | 4.2 (3.0-5.9 X 10^7^) | 3.7 (2.6- 5.4 X 10^7^) | > 0.05 |
| IgM memory (CD27+IgM+) | 6.6 (4.8-9.0 X 10^6^) | 5.7 (4.3-7.5 X 10^6^) | > 0.05 |
| Class Switched (CD27-IgM-) | 1.3 (1.1-1.7 X 10^7^) | 1.3 (1.0-1.6 X 10^7^) | > 0.05 |
| Class Switched memory (CD27+IgM-) | 1.5 (1.2-2.0 X 10^7^) | 1.6 (1.2-2.1 X 10^7^) | > 0.05 |
| CD19+CD5+ | 2.2 (1.8-2.7 X 10^7^) | 2.2 (1.6-3.0 X 10^7^) | > 0.05 |
| CD19+CD5- | 5.0 (3.5-7.0 X 10^7^) | 4.3 (2.9-6.3 X 10^7^) | > 0.05 |
