# Supplementary material for: The effect of pneumococcal immunization on total and antigen-specific B cells in patients with severe chronic kidney disease
Source: BMC Immunol. 2019 Nov 12;20:41. doi: 10.1186/s12865-019-0325-9 (PMC6849264; doi:10.1186/s12865-019-0325-9)
Supplement: Supplementary file 2 — Additional file 2: Table S2. Correlation of absolute numbers and proportions of B cells and B-cell subpopulations pre- and 7 days post-immunization. Correlation between pre- and post-immunization absolute numbers or proportions of total B cells (CD19+), naïve (CD27-IgM+), IgM memory (CD27 + IgM+), class switched (CD27-IgM-), class switched memory (CD27 + IgM), CD5+ and CD5- B cells. [file 12865_2019_325_MOESM2_ESM.docx]

|  | Correlation of absolute numbers of cells | | Correlation of proportions of cells | |
| --- | --- | --- | --- | --- |
| B cells | r | p | r | p |
| Total B cells (CD19+) | 0.6275 Spearman | < 0.0001 | 0.3414 Spearman | > 0.05 |
| Naïve (CD27-IgM+) | 0.8230 Spearman | < 0.0001 | 0.8680 Pearson | < 0.0001 |
| IgM memory (CD27+IgM+) | 0.7601 Spearman | < 0.0001 | 0.8937 Pearson | < 0.0001 |
| Class Switched (CD27-IgM-) | 0.0222 Pearson | 0.9090 | 0.5030 Spearman | 0.0064 |
| Class Switched memory (CD27+IgM-) | 0.6759 Spearman | < 0.0001 | 0.8492 Spearman | < 0.0001 |
| CD19+CD5+ | 0.6940 Spearman | < 0.0001 | 0.7384 Spearman | < 0.0001 |
| CD19+CD5- | 0.4501 Spearman | 0.0126 | 0.7259 Spearman | < 0.0001 |
