# Supplementary material for: The effect of pneumococcal immunization on total and antigen-specific B cells in patients with severe chronic kidney disease
Source: BMC Immunol. 2019 Nov 12;20:41. doi: 10.1186/s12865-019-0325-9 (PMC6849264; doi:10.1186/s12865-019-0325-9)
Supplement: Supplementary file 3 — Additional file 3: Table S3. Absolute numbers of lymphocytes and B cells in patients with severe chronic kidney disease that are pneumococcal vaccine naïve or previously immunized with PPV23 > 1 year ago. Absolute numbers of lymphocytes and B cells (geometric means, GM with 95% confidence intervals, CI) in severe chronic kidney disease patients that are pneumococcal vaccine naïve (n = 14) or previously immunized with PPV23 > 1 year ago (n = 19) pre- and 7 days post-immunization with PCV13. [file 12865_2019_325_MOESM3_ESM.docx]

| Cells | PPV23 naïve  Absolute number of cells/L (GM, CI) | PPV23 > 1 year ago Absolute number of cells/L (GM, CI) | p value |
| --- | --- | --- | --- |
| Total lymphocytes pre-immunization | 1.0 (0.8-1.4 X 10^9^) | 0.9 (0.8-1.1 X 10^9^) | > 0.05 |
| Total lymphocytes 7 days post-immunization | 1.0 (0.7-1.3 X 10^9^) | 1.0 (0.8-1.2 X 10^9^) | > 0.05 |
| Total B cells pre-immunization | 9.9 (7.0-13.9 X 10^7^) | 8.3 (6.1-11.2 X 10^7^) | > 0.05 |
| Total B cells 7 days post-immunization | 9.3 (6.7-12.8 X 10^7^) | 7.9 (5.7-10.9 X 10^7^) | > 0.05 |
