# Supplementary material for: The effect of pneumococcal immunization on total and antigen-specific B cells in patients with severe chronic kidney disease
Source: BMC Immunol. 2019 Nov 12;20:41. doi: 10.1186/s12865-019-0325-9 (PMC6849264; doi:10.1186/s12865-019-0325-9)
Supplement: Supplementary file 4 — Additional file 4: Table S4. Concentrations and fold change of pneumococcal 6B and 14 IgG antibodies pre- and 28 days post-immunization. Geometric mean concentration (GMC) with 95% confidence intervals (CI) in severe chronic kidney disease patients who are pneumococcal vaccine naïve (n = 22) or previously immunized with PPV23 > 1 year ago (n = 34) pre- and 28 days post-immunization with PCV13. The fold change represents the response to PCV13 immunization. * compares day 0 pre-immunization and day 28 post-immunization antibody concentrations for each serotype and patient group, + compares the pre-immunization concentrations between patient groups, # compares the fold change for each serotype between groups. Statistical significance determined by Mann- Whitney U test, * p < 0.05, ** p < 0.01. [file 12865_2019_325_MOESM4_ESM.docx]

| Serotype | Group | GMC, 95% CI (µg/ mL)  Day 0 pre-immunization | GMC, 95% CI (µg/ mL)  Day 28 post-immunization | Fold change |
| --- | --- | --- | --- | --- |
| 6B | PPV23 naive | 0.9 (0.6- 1.4) | 2.1 (1.4- 3.1) * | 2.8 |
|  | PPV23 > 1 year ago | 1.1 (0.7- 1.6) | 2.4 (1.4- 3.9) * | 2.3 |
| 14 | PPV23 naive | 2.8 (1.9- 4.2) + | 8.0 (4.5- 14.4) ** | 1.9 # |
|  | PPV23 > 1 year ago | 5.0 (3.0- 8.1) | 8.1 (5.0- 13.3) | 1.3 |
